# Combination of early rhythm control and healthy lifestyle on the risk of stroke in elderly patients with new-onset atrial fibrillation: a nationwide population-based cohort study

**DOI:** 10.3389/fcvm.2024.1346414

**Published:** 2024-02-15

**Authors:** Woo-Hyun Lim, So-Ryoung Lee, Eue-Keun Choi, Seung-Woo Lee, Kyung-Do Han, Seil Oh, Gregory Y. H. Lip

**Affiliations:** ^1^ Department of Internal Medicine, Seoul National University Boramae Medical Center, Seoul, Republic of Korea; ^2^ Department of Internal Medicine, Seoul National University Hospital, Seoul, Republic of Korea; ^3^ Department of Internal Medicine, Seoul National University College of Medicine, Seoul, Republic of Korea; ^4^ Department of Medical Statistics, College of Medicine, Catholic University of Korea, Seoul, Republic of Korea; ^5^ Statistics and Actuarial Science, Soongsil University, Seoul, Republic of Korea; ^6^ Liverpool Centre for Cardiovascular Science at University of Liverpool, Liverpool John Moores University and Liverpool Chest and Heart Hospital, Liverpool, United Kingdom; ^7^ Department of Clinical Medicine, Aalborg University, Aalborg, Denmark

**Keywords:** atrial fibrillation, rhythm control, lifestyle modification, stroke, elderly

## Abstract

**Background:**

The impact of early rhythm control (ERC) combined with healthy lifestyle (HLS) on the risk of ischemic stroke in elderly patients with atrial fibrillation (AF) remains unaddressed.

**Objective:**

To evaluate the impact of combined ERC and HLS on the risk of stroke in elderly patients with new-onset AF.

**Methods:**

Using the Korean National Health Insurance Service database, we included patients aged ≥75 years with new-onset AF from January 2009 to December 2016 (*n* = 41,315). Patients who received rhythm control therapy within 2 years of AF diagnosis were defined as the ERC group. Non-smoking, non-to-mild alcohol consumption (<105 g/week), and regular exercise were defined as HLS. Subjects were categorized into four groups: group 1 (without ERC and HLS, *n* = 25,093), 2 (HLS alone, *n* = 8,351), 3 (ERC alone, *n* = 5,565), and 4 (both ERC and HLS, *n* = 2,306). We assessed the incidence of ischemic stroke as the primary outcome, along with admissions for heart failure, all-cause death, and the composite of ischemic stroke, admission for heart failure, and all-cause death.

**Results:**

Median follow-up duration of the study cohort was 3.4 years. After adjusting for multiple variables, groups 2 and 3 were associated with a lower stroke risk (adjusted hazard ratio [aHR]: 95% confidence interval [CI]: 0.867, 0.794–0.948 and 0.713, 0.637–0.798, respectively) than that of group 1. Compared to Group 1, group 4 showed the lowest stroke risk (aHR: 0.694, 95% CI: 0.586–0.822) among all groups, followed by group 3 (0.713, 0.637–0.798) and group 2 (0.857, 0.794–0.948), respectively. Group 4 was associated with the lowest risk of all-cause death (aHR: 0.680, 95% CI: 0.613–0.754) and the composite outcome (aHR: 0.708, 95% CI: 0.649–0.772).

**Conclusion:**

ERC and HLS were associated with a lower risk of ischemic stroke in elderly patients with new-onset AF. Concurrently implementing ERC and maintaining HLS was associated with the lowest risk of death and the composite outcome, with a modest synergistic effect on stroke prevention.

## Introduction

Atrial fibrillation (AF) is the most prevalent sustained arrhythmia in the elderly (1). Its incidence increases with age, and individuals older than 80 years have a prevalence of AF exceeding 10% (2, 3). Recent data reports that the projected lifetime risk of AF in individuals older than 40 years has increased to 1 in 3 (4). AF is strongly associated with a 5-fold increased risk of ischemic stroke and a 3-fold increase in the risk of heart failure (5, 6). Considering the associations between AF and cardiovascular morbidity and mortality, this arrhythmia imposes a substantial burden on the public health of the elderly.

Guidelines have emphasized the need for an integrated approach to AF management to achieve better clinical outcomes (7, 8). Alongside appropriate anticoagulation therapy and symptom control, the management of cardiovascular risk factors and comorbidities, including lifestyle modification, is crucial (9, 10). Recent studies have demonstrated that early rhythm control (ERC) therapy within 1 year of AF diagnosis can effectively reduce the risk of adverse cardiovascular outcomes compared to conventional care in patients with AF (11, 12). Nevertheless, lifestyle modification is not routinely recommended for elderly patients (13) and older patients with AF are less likely to be offered rhythm control therapy (14). Furthermore, whether ERC therapy and lifestyle modification retain their effectiveness in the elderly remains uncertain.

In this study, we aimed to evaluate the impact of ERC therapy and a healthy lifestyle (HLS) and the combination of both on the risk of ischemic stroke, admission for heart failure, all-cause mortality, and composite clinical outcome in patients with AF aged 75 years and older.

## Methods

We conducted an observational cohort study using data from a nationwide claims database administered by the Korean National Health Insurance Service (NHIS). The NHIS provides universal coverage, guaranteeing that the extracted data is considered representative of the entire Korean population. To gather information on lifestyle behaviors, we accessed the health check-up database linked to the NHIS database (15, 16). This database is maintained by the National Health Insurance Corporation and includes biennial health screening check-up data for all Korean adults. Integration of this information with the NHIS database is a well-established practice. As of 2016, the adult health examination rate was estimated to be 85%. It is possible to access the data through the NHIS National Health Insurance Sharing Service homepage (http://nhiss.nhis.or.kr). Applications for access to NHIS data are evaluated by the research support inquiry committee. Upon approval, authorized researchers are granted access to the raw data at designated locations. This study was approved by the Institutional Review Board of Seoul National University Hospital (E-2312-019-1488). The requirement for obtaining informed consent from study participants was waived due to the stringent confidentiality guidelines that encrypt personal identification information during cohort generation.

### Study population

**Figure 1:**
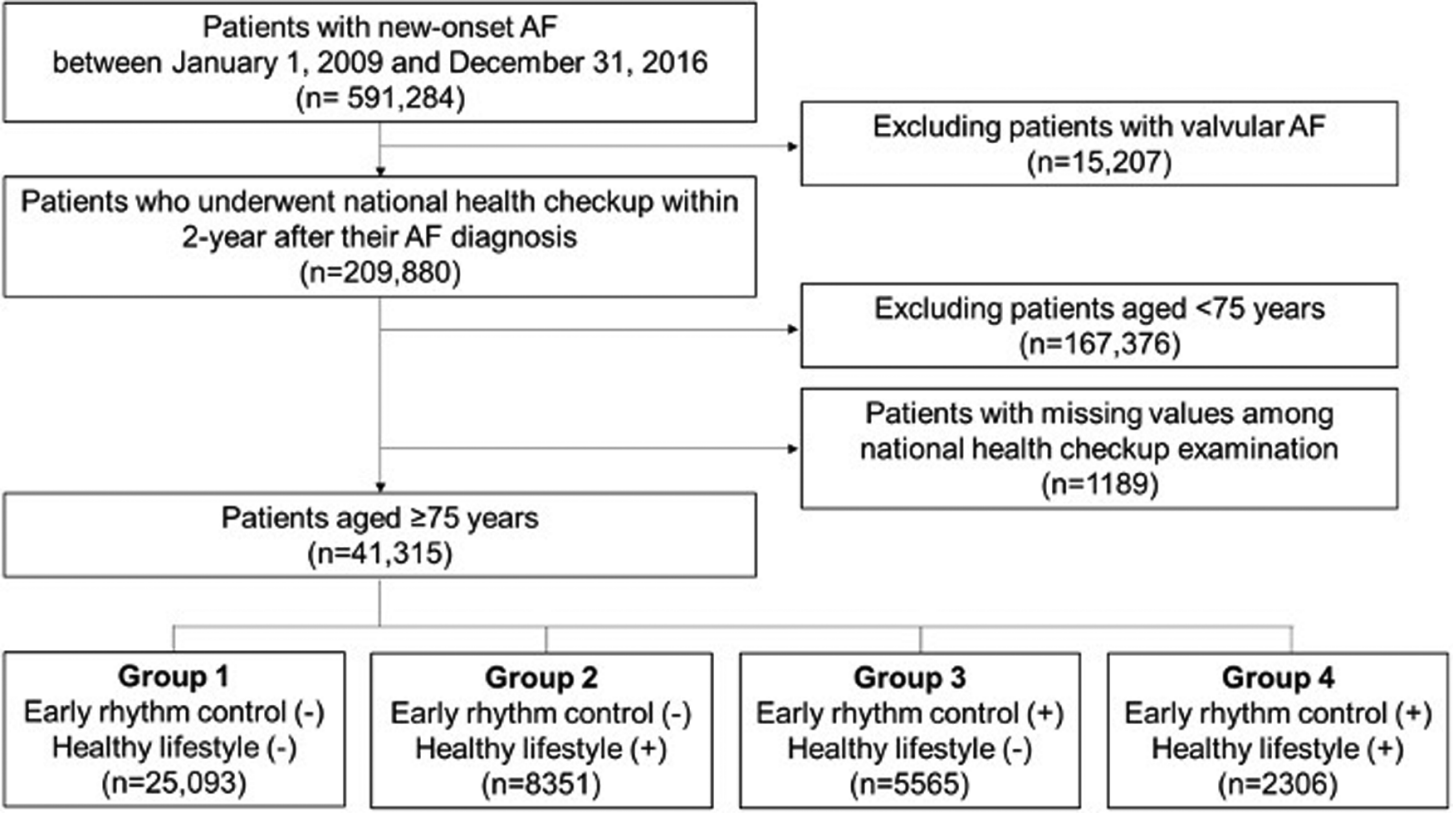
Study flow. AF, atrial fibrillation.

We identified patients who were newly diagnosed with AF between January 1, 2009, and December 31, 2016. For inclusion in this study, we selected patients who had undergone a national health check-up within 2 years of their AF diagnosis because most health check-ups were offered biennially. Figure 1 illustrates the enrollment process and Supplementary Figure S1 shows the time-line graph of data collection.

### Definition of early rhythm control, healthy lifestyle, and the combination of both

Rhythm control therapy was defined as the prescription of antiarrhythmic drugs (AAD, class Ic, or class III), direct current cardioversion (DCC), or AF catheter ablation (Supplementary Table S1). Patients who received rhythm control therapy within 2 years of new-onset AF were categorized as the ERC group. Lifestyle behaviors, including smoking, drinking, and physical activity, were assessed using self-reported questionnaires during health check-ups. HLS behaviors were defined as non-current smoking, non-to-mild alcohol consumption (<105 g/week), and performing regular exercise (17–19). Regular exercise was further specified as moderate-intensity physical activity (e.g., brisk-pace walking, tennis doubles, or cycling leisurely for more than 30 min) or vigorous-intensity physical activity (e.g., running, climbing, fast cycling, or performing aerobics for more than 20 min) at least once a week (17). In this study, patients displaying all three HLS behaviors (non-smoker, non-to-mild drinker, and regular exerciser) were categorized as the HLS group. Using a two-by-two factorial design, patients were categorized into the following four groups: group 1, without both ERC and HLS; group 2, HLS alone; group 3, ERC alone; and group 4, both ERC and HLS (Figure 1).

### Covariates

Patient age, sex, and comorbidities were determined using diagnostic codes, prescription records, inpatient and outpatient hospital visits, and health check-up results. Detailed definitions of comorbidities are provided in Supplementary Table S1. Current medications, including AADs, oral anticoagulants (OACs; e.g., warfarin and direct oral anticoagulants), antiplatelet agents, statins, beta-blockers, calcium channel blockers, digoxin, renin–angiotensin system blockers, and diuretics, were identified based on prescription records. The body mass index (BMI), blood pressure, and estimated glomerular filtration rate were obtained from baseline health check-ups. Using these baseline covariates, the CHA_2_DS_2_-VASc score and Charlson comorbidity index (CCI) were calculated on the basis of patients' comorbidities and medical history (Supplementary Tables S1, S2) (20).

Lifestyle behaviors were evaluated through self-reported questionnaires during national health check-ups. The HLS behavior score was computed by assigning 1 point each for non-current smoking, non-to-mild drinking, and regular exercise. The bottom 20% of the household income among all NHIS subscribers was classified as low-income.

### Study outcome and follow-up

During the follow-up period, we evaluated outcomes such as ischemic stroke, admission for heart failure, all-cause death, and the composite of these three outcomes. A detailed definition of these outcomes is provided in Supplementary Table S1. Follow-up for study outcomes commenced on the date of the baseline health examination, and patients were monitored until the occurrence of the primary outcome, death, or the end of the study (December 31, 2018), whichever came first. In this study, a detailed investigation into the specific causes of death was not conducted. Nevertheless, we conducted an exploratory analysis for cardiovascular (CV) deaths by defining them arbitrarily as deaths associated with an international classification of disease-10 I code.

### Statistical analysis

Continuous variables are presented as means and standard deviations, whereas categorical variables are presented as numbers and percentages. We used one-way analysis of variance to compare continuous variables and the chi-square test for categorical variables to examine the significance of differences among the four groups. Incidence rates (IR) for ischemic stroke as the primary outcome, along with admissions for heart failure, all-cause death, and the composite of ischemic stroke, admission for heart failure, and all-cause death were calculated as the number of events per 100 person-years (PY). We used Cox proportional hazard regression models to estimate hazard ratios (HRs) and the corresponding 95% confidence intervals (CIs). Initially, unadjusted and age-and sex-adjusted HRs were assessed (model 1 and model 2 respectively). Subsequently, a multivariate Cox analysis was conducted, adjusting for age, sex, all comorbidities, CHA_2_DS_2_-VASc score, CCI, all medications, BMI, systolic blood pressure, and income (model 3). The criteria for selecting covariates for Model 3 were determined based on all known factors influencing cardiovascular outcomes in patients with AF, along with baseline covariates included in this study (Table 1) that showed a significant difference (*p*-value < 0.1) between groups. The evaluation of multicollinearity was conducted using the variance inflation factor, indicating the absence of noteworthy collinearity among the incorporated covariates (Variance inflation factor = 1.00–1.70).

**Table 1:** Baseline characteristics.

	Total	Group 1 ERC (–), HLS (–)	Group 2 ERC (–), HLS (+)	Group 3 ERC (+), HLS (–)	Group 4 ERC (+) and HLS (+)	*P*-value
Number	41,315	25,093	8,351	5,565	2,306	
Duration from AF diagnosis to rhythm control, days						
Mean ± SD, days	28.0 ± 84.1	–	–	29.4 ± 87.7	24.6 ± 74.4	0.022
Median (IQR), days	0 (0–8)	–	–	0 (0–7)	0 (0–10)	0.009
Rhythm control						
Anti-arrhythmic agents	7,836 (19.0)	0 (0)	0 (0)	5,539 (99.5)	2,297 (99.6)	<0.001
Class Ic	3,906 (9.5)	0 (0)	0 (0)	2,686 (48.3)	1,220 (52.9)	<0.001
Class III	4,555 (11.0)	0 (0)	0 (0)	3,288 (59.1)	1,267 (54.9)	<0.001
Direct current cardioversion	300 (0.7)	0 (0)	0 (0)	201 (3.6)	99 (4.3)	<0.001
AF catheter ablation	59 (0.1)	0 (0)	0 (0)	35 (0.6)	24 (1.0)	<0.001
Healthy lifestyle behavior score						<0.001
** **0	695 (1.7)	579 (2.3)	0 (0)	116 (2.1)	0 (0)	
** **1	5,353 (13.0)	3,603 (14.4)	766 (9.2)	792 (14.2)	192 (8.3)	
** **2	31,399 (76.0)	20,911 (83.3)	4,628 (55.4)	4,657 (83.7)	1,203 (52.2)	
** **3	3,868 (9.4)	0 (0)	2,957 (35.4)	0 (0)	911 (39.5)	
Age, years	79.8 ± 3.9	80.1 ± 4.1	79.1 ± 3.4	79.5 ± 3.6	78.8 ± 3.2	<0.001
Men	20,442 (49.5)	11,539 (46.0)	4,805 (57.5)	2,641 (47.5)	1,457 (63.2)	<0.001
CHA_2_DS_2_-VASc	5.5 ± 1.5	5.6 ± 1.5	5.3 ± 1.5	5.6 ± 1.6	5.2 ± 1.6	<0.001
** **2	494 (1.2)	273 (1.1)	131 (1.6)	60 (1.1)	30 (1.3)	0.004
** **≥3	40,821 (98.8)	24,820 (98.9)	8,220 (98.4)	5,505 (98.9)	2,276 (98.7)	
CCI	4.1 ± 2.4	4.1 ± 2.4	3.9 ± 2.3	4.2 ± 2.4	4.0 ± 2.4	<0.001
Hypertension	38,472 (93.1)	23,382 (93.2)	7,730 (92.6)	5,208 (93.6)	2,152 (93.3)	0.102
Diabetes mellitus	11,425 (27.7)	7,015 (28.0)	2,236 (26.8)	1,563 (28.1)	611 (26.5)	0.092
Dyslipidemia	19,614 (47.5)	11,428 (45.5)	4,063 (48.65)	2,909 (52.3)	1,214 (52.7)	<0.001
Heart failure	20,104 (48.7)	12,316 (49.1)	3,626 (43.42)	3,053 (54.9)	1,109 (48.1)	<0.001
Prior ischemic stroke	17,524 (42.4)	11,110 (44.3)	3,280 (39.3)	2,271 (40.8)	863 (37.4)	<0.001
Prior ICH	609 (1.5)	378 (1.5)	106 (1.3)	97 (1.7)	28 (1.2)	0.092
Prior myocardial infarction	6,348 (15.4)	3,747 (14.9)	1,140 (13.7)	1,063 (19.1)	398 (17.3)	<0.001
Peripheral artery disease	12,460 (30.2)	7,681 (30.6)	2,459 (29.5)	1,666 (29.9)	654 (28.4)	0.043
COPD	12,781 (30.94)	7,933 (31.61)	2,358 (28.24)	1,803 (32.4)	687 (29.8)	<0.001
Cancer	2,600 (6.3)	1,494 (6.0)	599 (7.2)	329 (5.9)	178 (7.7)	<0.001
Chronic liver disease	5,887 (14.3)	3,609 (14.4)	1,100 (13.2)	863 (15.5)	315 (13.7)	0.001
Chronic kidney disease	13,347 (32.3)	8,230 (32.8)	2,387 (28.6)	1,985 (35.7)	745 (32.3)	<0.001
Osteoporosis	12,509 (30.3)	7,987 (31.8)	2,206 (26.4)	1,729 (31.1)	587 (25.5)	<0.001
Hyperthyroidism	2,763 (6.7)	1,575 (6.3)	540 (6.5)	479 (8.6)	169 (7.3)	<0.001
Hypothyroidism	3,722 (9.0)	2,146 (8.6)	688 (8.2)	614 (11.0)	274 (11.9)	<0.001
Sleep apnea	45 (0.1)	25 (0.1)	11 (0.1)	5 (0.1)	4 (0.2)	0.644
Body mass index (kg/m^2^)	23.5 ± 3.4	23.4 ± 3.5	23.8 ± 3.2	23.5 ± 3.4	23.8 ± 3.1	<0.001
Body mass index ≥25 kg/m^2^	13,179 (31.9)	7,767 (31.0)	2,860 (34.3)	1,777 (31.9)	775 (33.6)	<0.001
SBP (mmHg)	128.9 ± 16.8	128.8 ± 17.0	128.9 ± 16.1	128.6 ± 17.1	129.6 ± 15.7	0.138
DBP (mmHg)	76.7 ± 10.7	76.9 ± 10.8	76.9 ± 10.5	75.7 ± 10.8	75.9 ± 10.3	<0.001
Estimated GFR (ml/min)	70.0 ± 27.1	70.0 ± 26.8	71.1 ± 26.5	68.3 ± 29.5	69.4 ± 27.0	<0.001
Oral anticoagulants	21,568 (52.2)	12,353 (49.2)	4,368 (52.3)	3,339 (60.0)	1,508 (65.4)	<0.001
Warfarin	5,850 (14.2)	3,574 (14.2)	1,112 (13.3)	846 (15.2)	318 (13.8)	0.016
DOAC	15,718 (38.0)	8,779 (35.0)	3,256 (39.0)	2,493 (44.8)	1,190 (51.6)	<0.001
Antiplatelet agents	12,815 (31.0)	8,017 (32.0)	2,573 (30.8)	1,627 (29.2)	598 (25.9)	<0.001
Statin	8,307 (20.1)	4,895 (19.5)	1,704 (20.4)	1,177 (21.2)	531 (23.0)	<0.001
Beta-blocker	6,129 (14.8)	3,647 (14.5)	1,264 (15.1)	861 (15.5)	357 (15.5)	0.175
Non-DHP CCB	2,193 (5.3)	1,294 (5.2)	439 (5.3)	333 (6.0)	127 (5.5)	0.092
Digoxin	4,178 (10.1)	2,936 (11.7)	842 (10.1)	312 (5.6)	88 (3.8)	<0.001
DHP CCB	8,508 (20.6)	5,326 (21.2)	1,827 (21.9)	974 (17.5)	381 (16.5)	<0.001
ACEi/ARB	12,582 (30.5)	7,789 (31.0)	2,626 (31.5)	1,541 (27.7)	626 (27.2)	<0.001
Diuretics	11,664 (28.2)	7,519 (30.0)	2,187 (26.1)	1,472 (26.5)	486 (21.1)	<0.001
Low income	6,357 (15.4)	4,026 (16.0)	1,212 (14.5)	843 (15.2)	276 (12.0)	<0.001

ACEi, angiotensin-converting enzyme inhibitor; AF, atrial fibrillation; ARB angiotensin receptor blocker; CCB, calcium channel blocker; CCI, Charlson comorbidity index; COPD, chronic obstructive pulmonary disease; DHP, dihydropyridine; DBP, diastolic blood pressure; DOAC, direct oral anticoagulant; ERC, early rhythm control; GFR, glomerular filtration rate; HLS, healthy lifestyle; ICH, intracranial hemorrhage; IQR, interquartile range; SBP, systolic blood pressure; SD, standard deviation.

To assess the impact of adding ERC to HLS and adding HLS to ERC, two sets of comparisons were performed as follows: (1) groups 2 vs. 4 to analyse the impact of adding ERC on HLS; and (2) groups 3 vs. 4 to evaluate the impact of adding HLS on ERC. Propensity score (PS) weighting was applied using stabilized weights calculated from the PS to balance differences between the two groups (21). All covariates, including those in the final multivariate Cox analysis model, were used in the PS calculation. After PS weighting, we evaluated the absolute standardized differences (ASD) of covariates for all baseline variables to confirm the balance between the two groups (22). An ASD exceeding 0.1 indicates an imbalance in a covariate. We evaluated weighted IRs (per 100-PY) and weighted cumulative incidence curves using the Kaplan–Meier method with the log-rank test for the study outcomes. The HR for the study outcomes of the two groups was assessed using weighted Cox proportional hazard regression models with PS weighting. The reference groups in the two comparison sets were groups 2 and 3.

Because OAC therapy significantly affects the risk of ischemic stroke, we conducted a subgroup analysis according to the use or non-use of OAC to determine whether there was an interaction in the effect of ERC, HLS, and their combination, depending on OAC use.

All *p*-values were two-sided, and statistical significance was defined as a *p*-value < 0.05. We conducted statistical analyses using SAS version 9.4 (SAS Institute, Cary, NC).

## Results

### Baseline characteristics

A total of 41,315 patients were finally included (Figure 1). The mean age of the patients was 79.8 ± 3.9 years, the mean CHA_2_DS_2_-VASc score was 5.5 ± 1.5, and 98.8% of patients had a CHA_2_DS_2_-VASc score of ≥3. The baseline characteristics of the total study population are presented in Table 1. Among the total study population, 19% (groups 3 and 4) received ERC therapy. The mean duration from AF diagnosis to rhythm control was 28.0 ± 84.1 days, and 98.0% of these patients received ERC within 1 year of AF diagnosis. Among those receiving ERC, 99.6% were prescribed AAD, 3.8% received DC cardioversion, and 0.1% underwent AF catheter ablation. According to the prespecified definition, 25,093, 8,351, 5,565, and 2,306 patients were classified into groups 1, 2, 3, and 4, respectively (Figure 1). The baseline characteristics of each group are described in Table 1.

### Ischemic stroke, heart failure, all-cause mortality, and composite outcome

**Figure 2:**
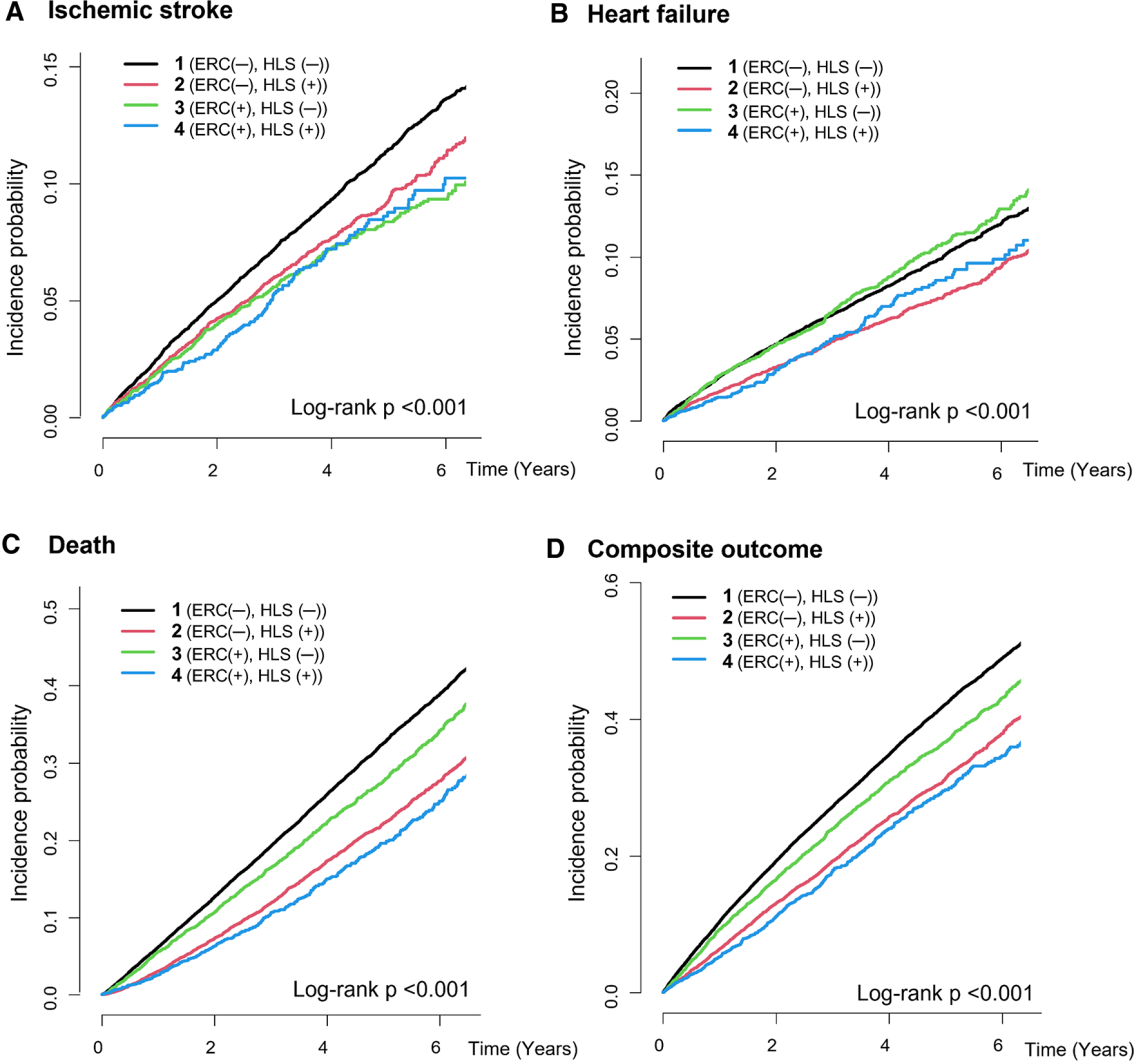
Cumulative incidence curves for the clinical outcomes of the study groups. (**A**) Ischemic stroke, (**B**) heart failure, (**C**) all-cause death, (**D**) composite outcome. ERC, early rhythm control; HLS, healthy lifestyle.

**Figure 3:**
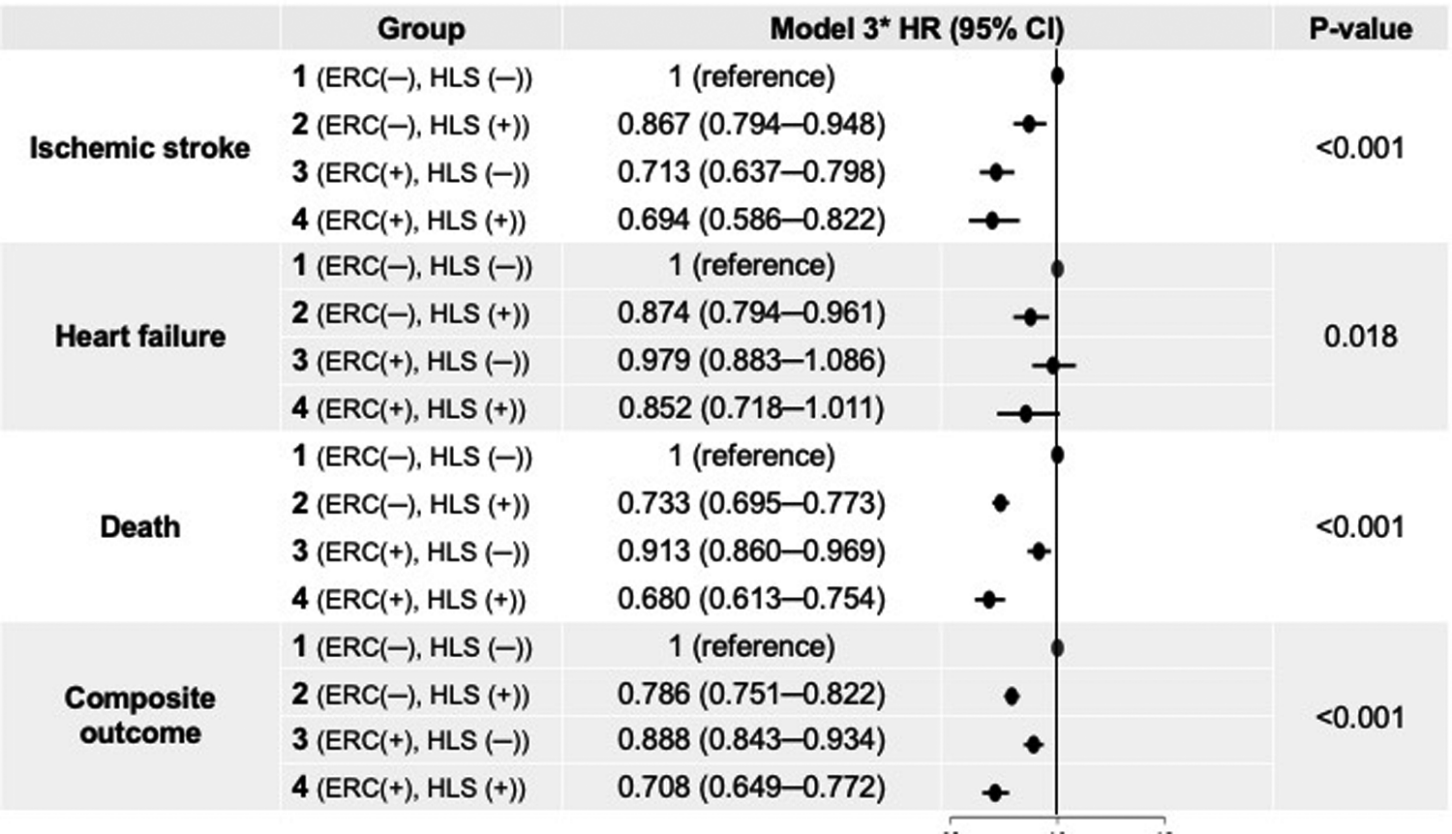
Adjusted hazard ratios for clinical outcomes of the study groups. *Model 3 was adjusted for age, sex, all comorbidities, CHA_2_DS_2_-VASc score, Charlson comorbidity index, all medications, body mass index, systolic blood pressure, and income. CI, confidence interval; ERC, early rhythm control; HLS, healthy lifestyle; HR, hazard ratio.

During a median 3.4-year follow-up (interquartile range: 2.0–5.4 years), 3,383 patients had ischemic stroke, 3,171 patients were admitted for heart failure, and 10,950 patients died (IR: 2.24, 2.09 and 6.94 per 100 PY, respectively). The crude numbers of events, crude incidence rates, and unadjusted and age- and sex-adjusted HRs for the clinical outcomes of the study groups are shown in Table 2. The Kaplan–Meier curves for the clinical outcomes of the study groups are presented in Figure 2. Figure 3 shows the adjusted HRs using model 3.

**Table 2:** Event numbers, incidence rates, unadjusted and adjusted hazard ratios for ischemic stroke, heart failure, death, composite outcome, and cardiovascular death.

	Number	Event	Incidence rate (Per 100 PY)	Model 1 HR (95% CI)	*p*-value	Model 2 HR (95% CI)	*p*-value	Model 3 HR (95% CI)	*p*-value
Ischemic stroke									
Group 1	25,093	2,239	2.45	1 (reference)	<0.001	1 (reference)	<0.001	1 (reference)	<0.001
Group 2	8,351	643	2.01	0.821 (0.752–0.897)		0.845 (0.774–0.923)		0.867 (0.794–0.948)	
Group 3	5,565	356	1.84	0.747 (0.668–0.836)		0.758 (0.678–0.848)		0.713 (0.637–0.798)	
Group 4	2,306	146	1.75	0.713 (0.603–0.843)		0.742 (0.627–0.878)		0.694 (0.586–0.822)	
Heart failure									
Group 1	25,093	2,038	2.22	1 (reference)	<0.001	1 (reference)	<0.001	1 (reference)	0.019
Group 2	8,351	542	1.68	0.759 (0.690–0.834)		0.831 (0.756–0.915)		0.874 (0.794–0.961)	
Group 3	5,565	447	2.32	1.043 (0.941–1.156)		1.088 (0.982–1.205)		0.979 (0.883–1.086)	
Group 4	2,306	144	1.71	0.773 (0.653–0.915)		0.884 (0.746–1.048)		0.852 (0.718–1.011)	
Death									
Group 1	25,093	7,552	7.89	1 (reference)	<0.001	1 (reference)	<0.001	1 (reference)	<0.001
Group 2	8,351	1,696	5.09	0.642 (0.609–0.677)		0.687 (0.651–0.724)		0.733 (0.695–0.773)	
Group 3	5,565	1,316	6.56	0.844 (0.796–0.895)		0.900 (0.849–0.954)		0.913 (0.860–0.969)	
Group 4	2,306	386	4.45	0.571 (0.515–0.632)		0.615 (0.555–0.682)		0.680 (0.613–0.754)	
Composite outcome									
Group 1	25,093	9,677	11.03	1 (reference)	<0.001	1 (reference)	<0.001	1 (reference)	<0.001
Group 2	8,351	2,382	7.69	0.696 (0.665–0.728)		0.744 (0.711–0.778)		0.786 (0.751–0.822)	
Group 3	5,565	1,752	9.38	0.855 (0.812–0.899)		0.899 (0.855–0.946)		0.888 (0.843–0.934)	
Group 4	2,306	554	6.83	0.623 (0.572–0.679)		0.672 (0.617–0.733)		0.708 (0.649–0.772)	
Cardiovascular death^a^									
Group 1	25,093	2,716	2.84	1 (reference)	<0.001	1 (reference)	<0.001	1 (reference)	<0.001
Group 2	8,351	578	1.74	0.609 (0.556–0.666)		0.678 (0.620–0.743)		0.721 (0.658–0.789)	
Group 3	5,565	460	2.29	0.821 (0.743–0.906)		0.880 (0.797–0.971)		0.866 (0.784–0.957)	
Group 4	2,306	126	1.45	0.518 (0.433–0.619)		0.602 (0.503–0.720)		0.650 (0.543–0.778)	

Model 1: unadjusted.

Model 2: adjusted for age and sex.

Model 3: adjusted for age, sex, CHA_2_DS_2_-VASc score, Charlson comorbidity index, hypertension, diabetes mellitus, dyslipidemia, heart failure, prior ischemic stroke, prior myocardial infarction, peripheral artery disease, chronic obstructive pulmonary disease, cancer, chronic liver disease, chronic kidney disease, osteoporosis, hyperthyroidism, hypothyroidism, sleep apnea, body mass index, systolic blood pressure, oral anticoagulants, antiplatelet agents, statin, beta-blocker, non-dihydropyridine calcium channel blocker, digoxin, dihydropyridine calcium channel blocker, angiotensin-converting enzyme inhibitor/angiotensin receptor blocker, diuretics, and low income.

CI, confidence interval; HR, hazard ratio; PY, person-year.

Group 1, without both early rhythm control (ERC) and healthy lifestyle (HLS); Group 2, HLS alone; Group 3, ERC alone; and Group 4, both ERC and HLS.

^a^ Cardiovascular death was defined as a death with an ICD-10 I code.

After multivariate adjustment using model 3, group 2 (maintaining HLS alone) was associated with a lower risk of ischemic stroke by 13% compared to group 1. Group 3 (implementing ERC alone) was also associated with a lower risk of ischemic stroke, showing a numerically greater risk reduction than maintaining HLS alone (by 29% compared to group 1). Group 4, patients receiving ERC and maintaining HLS, showed the greatest risk reduction for ischemic stroke by 31% compared to group 1. For admission with heart failure, group 2 was associated with a lower risk than group 1; however, group 3 did not show a significant difference compared to group 1, while group 4 showed only a borderline significant trend toward a lower risk of admission for heart failure compared to group 1.

Groups 2, 3, and 4 were associated with significantly lower risk of all-cause death than group 1 by 27% (adjusted HR, 95% CI; 0.733, 0.695–0.773), 9% (0.913, 0.860–0.969), and 32% (0.680, 0.613–0.754), respectively. For the composite outcome of ischemic stroke, admission for heart failure, and death, group 4 showed the greatest risk reduction by 29% (0.708, 0.649–0.772) compared to group 1. Groups 2 and 3 were also associated with a lower risk of the composite outcome by 21% (0.786, 0.751–0.822) and 11% (0.888, 0.843–0.934), respectively.

### Exploratory analysis for CV death

Among all deaths, CV deaths accounted for 35.4%. Groups 2, 3, and 4 showed significantly lower risk of CV death compared to group 1, with reduction of 28% (adjusted HR, 95% CI; 0.721, 0.658–0.789), 13% (0.866, 0.784–0.957), and 35% (0.680, 0.543–0.778) (Table 2). The reduction in CV death among the groups was similar to that of all-cause death. In other word, the observed reduction in all-cause death within group 2 and group 4 was primarily driven by a decrease in CV mortality.

### Sensitivity analyses

To evaluate whether there was an additional association when adding ERC to HLS alone or adding HLS to ERC alone, PS weighting analyses were performed for group 2 vs. group 4 and group 3 vs. group 4.

After PS weighting, the baseline characteristics of the two groups were well balanced with ASD <0.1 in all covariates (Supplementary Tables S3, S4). Compared to group 2, group 4 showed numerically lower weighted IRs for ischemic stroke, admission for heart failure, death, and the composite outcome, but the weighted HRs were not statistically significant (Supplementary Table S5). Compared to group 3, group 4 was associated with a significantly lower risk of composite outcome (weighted HR, 95% CI; 0.857, 0.782–0.940), mainly driven by all-cause death (0.801, 0.719–0.893) (Supplementary Table S6). Regarding the risk of ischemic stroke and admission with heart failure, there were no significant differences between groups 3 and 4.

### Subgroup analyses based on oral anticoagulant use

Overall, trends in association with the risk of clinical outcomes were similarly observed in both subgroups (Supplementary Table S7), and there was no significant interaction among subgroups for admission for heart failure and death. For ischemic stroke, the association of HLS alone was more accentuated in the subgroup without OAC treatment, whereas the association of ERC was more accentuated in the subgroup with OAC treatment. Similar findings were observed for HRs of the composite outcome.

## Discussion

In this large-scale observational study involving elderly patients with new-onset AF, our key findings were as follows: (1) Both ERC alone and HLS alone were associated with a significantly lower risk of ischemic stroke in patients aged ≥75 years than in those without ERC and HLS. The beneficial effect of ERC alone was more pronounced than that of HLS alone; (2) for all-cause death, both ERC alone and HLS alone were associated with a lower risk compared to those without ERC and HLS; however, a greater HR reduction was observed in the HLS alone group than in the ERC alone group, which exhibited a more modest HR reduction; and (3) in elderly patients with new-onset AF, the greatest risk reduction for the composite outcome was observed in those with both ERC and HLS (by 29%), followed by those with HLS alone (by 21%), and those with ERC alone (by 11%) compared to those without ERC and HLS.

The Early Treatment of Atrial Fibrillation for Stroke Prevention Trial (EAST-AFNET 4) demonstrated that implementing ERC therapy within 1 year of AF diagnosis reduced the incidence of major cardiovascular adverse events, including stroke, by 35% compared to conventional care (11). Several subsequent observational studies validated the findings of the EAST-AFNET 4 trial (12, 23). However, the clinical benefit of ERC therapy seems to be less obvious in the elderly population with AF. According to the EAST-AFNET 4 trial, the effects of ERC were consistent across all age tertial groups. In contrast, Dickow et al. demonstrated that the effects of ERC were greater in patients aged <75 years (23). Our study showed a clear benefit of ERC therapy in elderly individuals in terms of lowering the risk of ischemic stroke or all-cause death.

Elderly patients with AF often present with multiple comorbidities and clinical complexity, which increase the risk of stroke and other adverse outcomes (24). Moreover, advanced age itself is a major risk factor for stroke (25). However, older patients with AF are less likely to receive optimal treatment. Although there are sufficient data to support the use of oral anticoagulants (OAC) in this population (26), older people are less likely to receive OAC (27). Similarly, older patients with AF tend to receive less rhythm control therapy (14), which can offer benefits such as improved symptom control, enhanced quality of life, and potentially reduced long-term complications, including stroke.

In our study, only 48% of the study subjects received anticoagulation therapy, whereas a mere 19% received ERC therapy. Furthermore, among those receiving ERC therapy, only 0.7% underwent AF catheter ablation. Hence, our study suggests that ERC therapy should be actively considered in elderly patients in addition to appropriate anticoagulation and comorbidities control.

Our study reflects real-world practices in the treatment of elderly patients with AF. Out of the study subjects, only 48% received anticoagulation therapy. Although guidelines strongly recommend anticoagulation for patients with AF aged 75 years and older, it is frequently omitted in those with relatively low CHA_2_DS_2_-VASc scores (7, 8). Even with the increasing use of direct oral anticoagulants, up to 50% of Korean patients with AF and an OAC indication are not prescribed OAC (28). Furthermore, a mere 19% received ERC therapy, and within this group, only 0.7% underwent AF catheter ablation. Subgroup analysis revealed that the benefits of ERC alone (group 3) were significant only in the OAC group, not in the no OAC group. Additionally, the advantages of the combining ERC and HLS were more pronounced in the OAC group compared to the no OAC group. Taken together, our study suggests that ERC and HLS should be actively considered in elderly AF patients under the premise of appropriate anticoagulation and comorbidity control.

Increasing evidence suggests that lifestyle modification plays a pivotal role in the comprehensive management of AF. Correcting unhealthy lifestyles such as cigarette smoking cessation, regular exercise, alcohol abstinence, and weight reduction is associated with a significant reduction in AF burden and maintenance of sinus rhythm (29–33). As a result, current guidelines emphasize a more holistic or integrated approach to AF management; this includes lifestyle modification (7, 8, 34), which is associated with improved clinical outcomes (35–37). We observed a significant association between HLS and a lower risk of ischemic stroke, heart failure admissions, and all-cause mortality in the elderly population, which is consistent with the results from several recent observational studies of the general population (17–19), clearly demonstrating the importance of HLS in AF management, even among the elderly.

Based on our results, the synergistic effect of ERC and HLS on the risk of ischemic stroke was relatively modest. Nevertheless, HLS appears to be associated with a more substantial reduction in the risk of all-cause mortality than ERC, and the most significant risk reduction for the composite outcome was observed in elderly patients with AF who both implemented ERC and kept HLS. One possible explanation for this observation is that the population-attributable risk of stroke associated with AF increases exponentially with advancing age (5). In elderly individuals, interventions such as anticoagulation and rhythm control therapy can effectively reduce the risk of stroke in the presence of AF. This interpretation is further supported by the finding that the risk reduction of ischemic stroke in the HLS-alone group was more pronounced in the subgroup without OAC treatment.

### Study limitations

This study has several limitations. First, given that the study used the NHIS database, it is important to acknowledge that information not available in the database could act as a confounding factor. Second, we failed to obtain information regarding the subjects' rhythm status and maintenance of sinus restoration as well as symptoms related to AF. Third, the definition of HLS was confined to smoking, drinking, and physical activity, which could be assessed using national health check-up data. We did not include several factors such as weight reduction, a healthy diet, or moderation in caffeine consumption, although we adjusted for BMI in the multivariate analysis. Moreover, while ERC represents a newly implemented treatment, the evaluation of lifestyle was based on each individual's pre-existing healthy lifestyle, which was assessed at the time of the new AF diagnosis. To interpret HLS as an intervention, prospective studies would be necessary. Fourth, although patients' lifestyles could change during follow-up, our analyses did not include the impact of maintaining and changes in components of HLS during the follow-up period on clinical outcomes. We believe that having and consistently maintaining healthier lifestyle will result in favorable outcomes. This aspect requires further investigation in future studies. Finally, based on current guidelines, anticoagulation therapy should be initiated in patients with AF aged >75 years, unless there is a clear contraindication (7, 8). Ideally, the rate of anticoagulation treatment should be close to 100%. However, in our study cohort, it was only 52%, which reflects actual practice in the real world (28). Therefore, we conducted subgroup analyses to examine the use of OAC treatment and identified an interaction between the use of OAC and stroke outcomes. Generalizing our findings to populations with higher anticoagulation rates may be challenging.

## Conclusion

ERC and HLS may individually be associated with a lower risk of ischemic stroke in elderly patients with new-onset AF. Concurrently implementing ERC and maintaining HLS was associated with the lowest risk of death and the composite outcome, with a modest synergistic effect on stroke prevention. An integrated approach that includes both ERC and HLS should be considered for achieving better clinical outcomes in elderly patients newly diagnosed with AF.

## Data Availability

Publicly available datasets were analyzed in this study. This data can be found here: http://nhiss.nhis.or.kr.
