# Supplementary material for: Combination of early rhythm control and healthy lifestyle on the risk of stroke in elderly patients with new-onset atrial fibrillation: a nationwide population-based cohort study
Source: Front Cardiovasc Med. 2024 Feb 15;11:1346414. doi: 10.3389/fcvm.2024.1346414 (PMC10902049; doi:10.3389/fcvm.2024.1346414)
Supplement: Supplementary file 1 [file Table1.docx]

**Supplementary Materials**

**Supplementary Table 1. Definition of covariates and outcomes**

**Supplementary Table 2. Operational definition of Charlson Comorbidity Index**

**Supplementary Table 3. Baseline characteristics of group 1 versus group 2 after propensity score weighting**

**Supplementary Table 4. Weighted event numbers, incidence rates, and weighted hazard ratios for group 2 versus group 4 after propensity score weighting**

**Supplementary Table 5. Baseline characteristics of group 3 versus group 4 after propensity score weighting**

**Supplementary Table 6. Weighted event numbers, incidence rates, and weighted hazard ratios for group 3 versus group 4 after propensity score weighting**

**Supplementary Table 7. Subgroup analyses according to oral anticoagulant use**

**Supplementary Table 1. Definition of covariates and outcomes**

| **Diagnosis** | **ICD-10-CM code and definition** | **Diagnostic definition** |
| --- | --- | --- |
| **Inclusion/exclusion criteria** |  |  |
| **Atrial fibrillation** | I48.0-48.4, I48.9 | Admission or outpatient department≥1 |
| **Valvular atrial fibrillation** | I05.0, I05.2, I05.9, Z95.2-Z95.4 | Admission or outpatient department≥1 |
| **Rhythm control strategy** |  |  |
| **Anti-arrhythmic drug** |  |  |
| **Class Ic** | Flecainide, pilsicainide, propafenone |  |
| **Class III** | Amiodarone, sotalol, dronedarone |  |
| **Direct current cardioversion** | Claim codes: M5880 |  |
| **AF catheter ablation** | Claim codes: M6542 or M6547 |  |
| **Performing regular exercise** |  |  |
|  | Moderate physical activity was defined as ≥30 min per day of brisk walking, dancing, or gardening. Vigorous physical activity was defined as ≥20 min per day of running fast, cycling, or aerobic. The number of moderate or vigorous physical activities per week was also collected from the questionnaire. Regular exercise was defined as performing a moderate physical activity or a vigorous physical activity at least 1 time per week. | |
| **Comorbidities** |  |  |
| **Hypertension** | I10-I13, I15; and minimum 1 prescription of anti-hypertensive drug (thiazide, loop diuretics, aldosterone antagonist, alpha-/beta-blocker, calcium-channel blocker, angiotensin-converting enzyme inhibitor, angiotensin II receptor blocker). | Admission≥1 or outpatient department≥1 |
|  | Or systolic/diastolic blood pressure ≥ 140/90 mmHg | Index health examination |
| **Diabetes mellitus** | E11-E14; and minimum 1 prescription of anti-diabetic drugs (sulfonylureas, metformin, meglitinides, thiazolidinediones, dipeptidyl peptidase-4 inhibitors, α-glucosidase inhibitors, and insulin). | Admission≥1 or outpatient department≥1 |
|  | Or fasting glucose level ≥ 126 mg/dL | Index health examination |
| **Dyslipidemia** | E78 | Admission or outpatient department≥1 |
|  | Or Total cholesterol ≥ 240 mg/dL | Index health examination |
| **Heart failure** | I50 | Admission or outpatient department≥1 |
| **Prior ischemic stroke** | I63, I64 | Admission or outpatient department≥1 |
| **Prior intracranial hemorrhage** | I60, I61, I62 | Admission or outpatient department≥1 |
| **Prior myocardial infarction** | I21, I22 | Admission or outpatient department≥1 |
| **Peripheral artery disease** | I70, I73 | Admission or outpatient department≥1 |
| **Chronic obstructive pulmonary disease** | J41-44 | Admission or outpatient department≥1 |
| **Cancer** | C00-97 and RID code (V193) | Admission or outpatient department≥1 |
| **Chronic liver disease** | B18, K70, K71, K72, K73, K74, K76.1 | Admission or outpatient department≥1 |
| **Chronic kidney disease** | Estimated glomerular filtration rate <60 ml/min/1.73m^2^ | Index health examination |
| **Osteoporosis** | M80, M81, M82 (except M82.0) | Admission or outpatient department≥1 |
| **Hyperthyroidism** | E05 | Admission or outpatient department≥1 |
| **Hypothyroidism** | E03 | Admission or outpatient department≥1 |
| **Sleep apnea** | G47.3 | Admission or outpatient department≥1 |
| **Scores** |  |  |
| **CHA_2_DS_2_-VASc score** | Heart failure (1 point), hypertension (1 point), age ≥75 years (2 points), diabetes (1 point), previous stroke/systemic embolism/transient ischemic attack (2 points), vascular disease (prior MI or PAD, 1 point) and female sex (1 point) | |
|  |  |  |
| **Charlson comorbidity index** | See supplementary Table 2 |  |
| **Clinical outcome** |  |  |
| **Ischemic stroke** | I63, I64 | Primary diagnosis, admission≥1 (≥3 days) and brain imaging (CT or MRI) ≥1 |
| **Heart failure** | I50 | Primary diagnosis, admission≥1 |
| **Composite outcome** | Ischemic stroke + hospitalization for heart failure + all-cause death | Each definition was described as above. |

Abbreviation: ICD, international classification of disease; CM, clinical modification.

**Supplementary Table 2. Operational definition of Charlson Comorbidity Index**

| **Category** | **Weights** | **Disease** | **ICD-10-CM code** |
| --- | --- | --- | --- |
| **Myocardial infarction** | 1 | Acute myocardial infarction | I21 |
|  |  | Subsequent myocardial infarction | I22 |
| **Congestive heart failure** | 1 | Heart Failure | I50 |
| **Peripheral vascular disease** | 1 | Atherosclerosis | I70 |
|  |  | Other peripheral vascular disease | I73 |
| **Cerebrovascular disease** | 1 | Transient cerebral ischemic attacks and related syndromes | G45 |
|  |  | Vascular syndromes of brain in cerebrovascular diseases | G46 |
|  |  | Retinal vascular occlusion | H34 |
|  |  | Cerebrovascular disease | I60-I69 |
| **Dementia** | 1 | Dementia in Alzheimer's disease | F00 |
|  |  | Vascular dementia | F01 |
|  |  | Dementia in other disease classified elsewhere | F02 |
|  |  | Unspecified dementia | F03 |
| **Chronic pulmonary disease** | 1 | Chronic lower respiratory diseases | J40-J47 |
|  |  | Lung disease due to external agents | J60-J67 |
| **Rheumatic disease**  **(connective tissue disorder)** | 1 | Rheumatoid arthritis with rheumatoid factor | M05 |
|  |  | Felty's syndrome | M05.0 |
|  |  | Rheumatoid lung disease with rheumatoid arthritis | M05.1 |
|  |  | Rheumatoid vasculitis with rheumatoid arthritis | M05.2 |
|  |  | Rheumatoid heart disease with rheumatoid arthritis | M05.3 |
|  |  | Rheumatoid myopathy with rheumatoid arthritis | M05.4 |
|  |  | Rheumatoid polyneuropathy with rheumatoid arthritis | M05.5 |
|  |  | Rheumatoid arthritis with involvement of other organs and systems | M05.6 |
|  |  | Rheumatoid arthritis with rheumatoid factor without organ or systems involvement | M05.7 |
|  |  | Other rheumatoid arthritis with rheumatoid factor | M05.8 |
|  |  | Rheumatoid arthritis without rheumatoid factor | M05.9 |
|  |  | Adult-onset Still's disease | M06.1 |
|  |  | Rheumatoid bursitis | M06.2 |
|  |  | Rheumatoid nodule | M06.3 |
|  |  | Inflammatory polyarthropathy | M06.4 |
|  |  | Other specified rheumatoid arthritis | M06.8 |
|  |  | Rheumatoid arthritis, unspecified | M06.9 |
|  |  | Giant cell arteritis with polymyalgia rheumatica | M31.5 |
|  |  | Systemic lupus erythematosus (SLE) | M32 |
|  |  | Drug-induced SLE | M32.0 |
|  |  | SLE with organ or system involvement | M32.1 |
|  |  | Other forms of SLE | M32.8 |
|  |  | SLE, unspecified | M32.9 |
|  |  | Dermatopolymyositis | M33 |
|  |  | Juvenile dermatomyositis | M33.0 |
|  |  | Other dermatomyositis | M33.1 |
|  |  | Polymyositis | M33.2 |
|  |  | Dermatopolymyositis, unspecified | M33.9 |
|  |  | Systemic sclerosis [scleroderma] | M34 |
|  |  | Progressive systemic sclerosis | M34.0 |
|  |  | CR(E)ST syndrome | M34.1 |
|  |  | Systemic sclerosis induced by drug and chemical | M34.2 |
|  |  | Other forms of systemic sclerosis | M34.8 |
|  |  | Systemic sclerosis, unspecified | M34.9 |
|  |  | Other overlap syndromes | M35.1 |
|  |  | Polymyalgia rheumatica | M35.3 |
|  |  | Dermato(poly)myositis in neoplastic disease | M36.0 |
| **Peptic ulcer disease** | 1 | Gastric ulcer | K25 |
|  |  | Duodenal ulcer | K26 |
|  |  | Peptic ulcer, site unspecified | K27 |
|  |  | Gastrojejunal ulcer | K28 |
| **Mild liver disease** | 1 | Chronic viral hepatitis | B18 |
|  |  | Alcoholic fatty liver | K70.0- K70.3, K70.9 |
|  |  | Alcoholic hepatitis |  |
|  |  | Alcoholic fibrosis and sclerosis of liver |  |
|  |  | Alcoholic cirrhosis of liver |  |
|  |  | Alcoholic liver disease, unspecified |  |
|  |  | Toxic liver disease with chronic persistent hepatitis | K71.3- K71.5, K71.7 |
|  |  | Toxic liver disease with chronic lobular hepatitis |  |
|  |  | Toxic liver disease with chronic active hepatitis |  |
|  |  | Toxic liver disease with fibrosis and cirrhosis of liver |  |
|  |  | Chronic hepatitis, not elsewhere classified | K73 |
|  |  | Fibrosis and cirrhosis of liver | K74 |
|  |  | Fatty (change of) liver, not elsewhere classified | K76.0-K76.4, K76.8, K76.9 |
|  |  | Nonalcoholic fatty liver disease |  |
|  |  | Central hemorrhagic necrosis of liver |  |
|  |  | Infarction of liver |  |
|  |  | Hepatic angiomatosis |  |
|  |  | Other specified disease of liver |  |
|  |  | Simple cyst of liver |  |
|  |  | Focal nodular hyperplasia of liver |  |
|  |  | Hepatoptosis |  |
|  |  | Liver disease, unspecified |  |
|  |  | Liver transplant status | Z94.4 |
| **Diabetes without chronic**  **complication** | 1 | with coma | E10.0, 10.1, 10.6, 10.8, 10.9 |
|  |  | with ketoacidosis | E11.0, 11.1, 11.6, 11.8, 11.9 |
|  |  | with other specified complications | E12.0, 12.1, 12.6, 12.8, 12.9 |
|  |  | with unspecified complications | E13.0, 13.1, 13.6, 13.8, 13.9 |
|  |  | without complications | E14.0, 14.1, 14.6, 14.8, 14.9 |
| **Diabetes with chronic**  **complication** | 2 | with renal complications | E10.2, 10.3, 10.4, 10.5, 10.7 |
|  |  | with ophthalmic complications | E11.2, 11.3, 11.4, 11.5, 11.7 |
|  |  | with neurologic complications | E12.2, 12.3, 12.4, 12.5, 12.7 |
|  |  | with peripheral circulatory complications | E13.2, 13.3, 13.4, 13.5, 13.7 |
|  |  | with multiple complications | E14.2, 14.3, 14.4, 14.5, 14.7 |
| **Hemi/paraplegia** | 2 | Tropical spastic paraplegia | G04.1 |
|  |  | Hereditary spastic paraplegia | G11.4 |
|  |  | Spastic quadriplegic cerebral palsy | G80.0 |
|  |  | Spastic diplegic cerebral palsy | G80.1 |
|  |  | Spastic hemiplegic cerebral palsy | G80.2 |
|  |  | Flaccid hemiplegia | G81.0 |
|  |  | Spastic hemiplegia | G81.1 |
|  |  | Hemiplegia, unspecified | G81.9 |
|  |  | Flaccid paraplegia | G82.0 |
|  |  | Spastic paraplegia | G82.1 |
|  |  | Paraplegia, unspecified | G82.2 |
|  |  | Flaccid tetraplegia | G82.3 |
|  |  | Spastic tetraplegia | G82.4 |
|  |  | Tetraplegia, unspecified | G82.5 |
|  |  | Diplegia of upper limbs | G83.0 |
|  |  | Paralytic syndrome, unspecified | G83.9 |
| **Renal disease** | 2 | Hypertensive renal disease | I12 |
|  |  | Hypertensive heart and renal disease with renal failure | I13.1 |
|  |  | Chronic nephritic syndrome | N03 |
|  |  | Unspecified nephritic syndrome | N05 |
|  |  | Chronic kidney disease | N18 |
|  |  | Unspecified kidney failure | N19 |
|  |  | Disorders resulting from impaired renal tubular function | N25 |
|  |  | Care involving dialysis | Z49 |
|  |  | Transplanted organ and tissue status - kidney | Z94.0 |
|  |  | Dependence on renal dialysis | Z99.2 |
| **Cancer** | 2 | Any tumor, malignant neoplasm | C00-76, C97 |
|  |  | Any tumor, in situ neoplasm | D00-09 |
|  |  | Any tumor, Benign neoplasm | D10-36 |
|  |  | Any tumor, Neoplasm of unknown behavior | D37-48 |
|  |  | Leukemia | C91-95 |
|  |  | Lymphoma | C81-86 |
| **Metastatic cancer** | 3 | Metastatic solid tumor | C77-80 |
| **Moderate to severe**  **liver disease** | 3 | Esophageal varices | I85 |
|  |  | Gastric varices | I86.4 |
|  |  | Esophageal varices without bleeding in diseases classified elsewhere | I98.2 |
|  |  | Alcoholic hepatic failure | K70.4 |
|  |  | Toxic liver disease with hepatic necrosis | K71.1 |
|  |  | Hepatic failure (acute/chronic) due to drugs |  |
|  |  | Chronic hepatic failure | K72.1, K72.9 |
|  |  | Hepatic failure, unspecified |  |
|  |  | Hepatic veno-occlusive disease | K76.5-K76.7 |
|  |  | Portal hypertension |  |
|  |  | Hepatorenal syndrome |  |
| **Human immunodeficiency**  **Virus (HIV)** | 6 | HIV disease resulting in infectious and parasitic diseases | B20 |
|  |  | HIV disease resulting in malignant neoplasm | B21 |
|  |  | HIV disease resulting in other specified diseases | B22 |
|  |  | HIV disease resulting in other conditions | B23 |

**Supplementary Table 3. Baseline characteristics of group 2 versus group 4 after propensity score weighting**

|  | **Group 2** | **Group 4** | **ASD** |
| --- | --- | --- | --- |
| **Number** | **8353** | **2296** |  |
| **Duration from AF diagnosis to rhythm control, days** |  |  |  |
| **Mean ± SD, days** | - | 24.8±76.4 | - |
| **Median (IQR), days** | - | 0 (0-8) | - |
| **Rhythm control** |  |  |  |
| **Anti-arrhythmic agents** | 0 (0) | 2288.1 (99.7) | - |
| **Class Ic** | 0 (0) | 1209.7 (52.7) | - |
| **Class III** | 0 (0) | 1251.3 (54.5) | - |
| **Direct current cardioversion** | 0 (0) | 78 (3.4) | - |
| **AF catheter ablation** | 0 (0) | 18.9 (0.82) | - |
| **Healthy lifestyle behavior score** |  |  |  |
| **0** | - | - | - |
| **1** | 776.6 (9.3) | 177.1 (7.7) | - |
| **2** | 4607.6 (55.2) | 1203.1 (52.4) | - |
| **3** | 2968.4 (35.5) | 915.8 (39.9) | - |
| **Age, years** | 79.0±3.4 | 79.0±3.4 | 0.006 |
| **Men** | 4909.9(58.78) | 1363.3 (59.4) | 0.012 |
| **CHA_2_DS_2_-VASc** | 5.3±1.5 | 5.3±1.6 | 0.003 |
| **2** | 128.8 (1.5) | 32.7 (1.4) |  |
| **≥3** | 8223.8 (98.5) | 2263.3 (98.6) |  |
| **CCI** | 3.9±2.3 | 3.9±2.4 | 0.006 |
| **Hypertension** | 7746.6 (92.7) | 2134.6 (93.0) | 0.008 |
| **Diabetes mellitus** | 2229.1 (26.7) | 615.3 (26.8) | 0.002 |
| **Dyslipidemia** | 4134.8 (49.5) | 1132.7 (49.3) | 0.003 |
| **Heart failure** | 3711.7 (44.4) | 1023.9 (44.6) | 0.003 |
| **Prior ischemic stroke** | 3249 (38.9) | 894.4 (39.0) | 0.001 |
| **Prior ICH** | 106.7 (1.3) | 29.9 (1.3) | 0.002 |
| **Prior myocardial infarction** | 1209.3 (14.5) | 343.4 (15.0) | 0.013 |
| **Peripheral artery disease** | 2441.3 (29.2) | 678.1 (29.5) | 0.006 |
| **COPD** | 2389.3 (28.6) | 658 (28.7) | 0.001 |
| **Cancer** | 610.3 (7.31) | 169.2 (7.4) | 0.002 |
| **Chronic liver disease** | 1110.8 (13.3) | 313.1 (13.6) | 0.009 |
| **Chronic kidney disease** | 2454.8 (29.4) | 674.9 (29.4) | <0.001 |
| **Osteoporosis** | 2185.9 (26.2) | 589.9 (25.7) | 0.010 |
| **Hyperthyroidism** | 557.3 (6.7) | 155.6 (6.8) | 0.004 |
| **Hypothyroidism** | 756.3 (9.1) | 210.7 (9.2) | 0.004 |
| **Sleep apnea** | 12 (0.1) | 3.5 (0.2) | 0.002 |
| **Body mass index (kg/m^2^)** | 23.8±3.2 | 23.8±3.1 | <0.001 |
| **Body mass index ≥25 kg/m^2^** | 2862.1 (34.3) | 772.4 (33.6) | 0.013 |
| **SBP (mmHg)** | 129.1±16.1 | 128.8±15.6 | 0.018 |
| **DBP (mmHg)** | 77.0±10.5 | 75.7±10.1 | 0.121 |
| **Estimated GFR (mL/min)** | 70.9±26.8 | 70.7±29.5 | 0.008 |
| **Oral anticoagulants** | 4607.1 (55.2) | 1274.3 (55.5) | 0.006 |
| **Warfarin** | 1170.3 (14.0) | 270.8 (11.8) | 0.066 |
| **DOAC** | 3436.8 (41.2) | 1003.5 (43.7) | 0.051 |
| **Antiplatelet agents** | 2486.6 (29.8) | 682.6 (29.7) | <0.001 |
| **Statin** | 1752.5 (21.0) | 493.8 (21.5) | 0.012 |
| **Beta-blocker** | 1267.7 (15.2) | 348.3 (15.2) | <0.001 |
| **Non-DHP CCB** | 444.4 (5.3) | 127.9 (5.6) | 0.011 |
| **Digoxin** | 728.3 (8.7) | 191.9 (8.4) | 0.013 |
| **DHP CCB** | 1729.8 (20.7) | 465.5 (20.3) | 0.010 |
| **ACEi/ARB** | 2548.6 (30.5) | 694.3 (30.2) | 0.005 |
| **Diuretics** | 2093 (25.1) | 565.7 (24.6) | 0.009 |
| **Low income** | 1168.7 (14.0) | 327.6 (14.3) | 0.007 |

Abbreviations: ACEi, angiotensin-converting enzyme inhibitor; AF, atrial fibrillation; ARB angiotensin receptor blocker; ASD, absolute standardized difference; CCB, calcium channel blocker; CCI, Charlson comorbidity index; COPD, chronic obstructive pulmonary disease; DHP, dihydropyridine; DBP, diastolic blood pressure; DOAC, direct oral anticoagulant; GFR, glomerular filtration rate; ICH, intracranial hemorrhage; IQR, interquartile range; SBP, systolic blood pressure; SD, standard deviation.

Group 2, healthy lifestyle (HLS) alone; and Group 4, both early rhythm control and HLS

**Supplementary Table 4. Baseline characteristics of group 3 versus group 4 after propensity score weighting**

|  | **Group 3** | **Group 4** | **ASD** |
| --- | --- | --- | --- |
| **Number** | 5563 | 2308 |  |
| **Duration from AF diagnosis to rhythm control, days** |  |  |  |
| **Mean ± SD, days** | 68.7±29.1 | 68.6±26.0 | 0.058 |
| **Median (IQR), days** | 0 (0-7) | 0 (0-9) |  |
| **Rhythm control** |  |  |  |
| **Anti-arrhythmic agents** | 5536.9 (99.5) | 2299.7 (99.6) | 0.017 |
| **Class Ic** | 2729.7 (49.1) | 1168 (50.6) | 0.030 |
| **Class III** | 3252.3 (58.5) | 1321.4 (57.3) | 0.024 |
| **Direct current cardioversion** | 212.2 (3.8) | 84.3 (3.7) | 0.008 |
| **AF catheter ablation** | 38.6 (0.7) | 19.5 (0.8) | 0.017 |
| **Healthy lifestyle behavior score** |  |  |  |
| **0** | 128.4 (2.3) | 0 (0) |  |
| **1** | 857 (15.4) | 167.7 (7.3) |  |
| **2** | 4577.9 (82.3) | 1243.2 (53.9) |  |
| **3** | 0 (0) | 897.2 (38.9) |  |
| **Age, years** | 29.6±87.8 | 24.8±75.4 | 0.015 |
| **Men** | 2893.2 (52.0) | 1197.1 (51.9) | 0.002 |
| **CHA_2_DS_2_-VASc** | 79.3±3.5 | 79.2±3.5 | 0.007 |
| **2** | 68.3 (1.2) | 24.9 (1.1) |  |
| **≥3** | 5495 (98.8) | 2283.1 (98.9) |  |
| **CCI** | 3.5±1.1 | 3.5±1.1 | 0.015 |
| **Hypertension** | 5200.2 (93.5) | 2153.6 (93.3) | 0.006 |
| **Diabetes mellitus** | 1540.1 (27.7) | 650.6 (28.2) | 0.011 |
| **Dyslipidemia** | 2912.1 (52.3) | 1203.5 (52.2) | 0.003 |
| **Heart failure** | 2943 (52.9) | 1229.3 (53.3) | 0.007 |
| **Prior ischemic stroke** | 2217.9 (39.9) | 923.2 (40) | 0.002 |
| **Prior ICH** | 93.9 (1.7) | 31.8 (1.4) | 0.025 |
| **Prior myocardial infarction** | 1034.6 (18.6) | 424.8 (18.4) | 0.004 |
| **Peripheral artery disease** | 1644.1 (29.6) | 691.4 (30.0) | 0.008 |
| **COPD** | 1762.5 (31.7) | 742.7 (32.2) | 0.010 |
| **Cancer** | 357.1 (6.4) | 146.2 (6.3) | 0.003 |
| **Chronic liver disease** | 837.5 (15.1) | 364.7 (15.8) | 0.020 |
| **Chronic kidney disease** | 1928.2 (34.7) | 794.9 (34.4) | 0.004 |
| **Osteoporosis** | 1644 (29.6) | 697.2 (30.2) | 0.014 |
| **Hyperthyroidism** | 459.3 (8.3) | 196.6 (8.5) | 0.009 |
| **Hypothyroidism** | 624.6 (11.2) | 255.9 (11.1) | 0.004 |
| **Sleep apnea** | 6.7 (0.1) | 2.8 (0.1) | <0.001 |
| **Body mass index (kg/m^2^)** | 4.2±2.4 | 4.2±2.5 | <0.001 |
| **Body mass index ≥25 kg/m^2^** | 1817.6 (32.7) | 736.8 (31.9) | 0.015 |
| **SBP (mmHg)** | 105.7±27.8 | 105.2±25.9 | 0.006 |
| **DBP (mmHg)** | 128.9±17.1 | 128.8±15.7 | 0.009 |
| **Estimated GFR (mL/min)** | 75.8±10.9 | 75.7±10.2 | 0.003 |
| **Oral anticoagulants** | 3429.5 (61.6) | 1434.1 (62.1) | 0.010 |
| **Warfarin** | 863.6 (15.5) | 310.7 (13.5) | 0.058 |
| **DOAC** | 2565.9 (46.1) | 1123.3 (48.7) | 0.051 |
| **Antiplatelet agents** | 1573 (28.3) | 650.8 (28.2) | 0.001 |
| **Statin** | 1208.8 (21.7) | 510.6 (22.1) | 0.009 |
| **Beta-blocker** | 859.8 (15.5) | 351.8 (15.2) | 0.005 |
| **Non-DHP CCB** | 323.9 (5.8) | 135 (5.9) | 0.001 |
| **Digoxin** | 283.9 (5.1) | 123.4 (5.4) | 0.010 |
| **DHP CCB** | 959.2 (17.2) | 395.4 (17.1) | 0.002 |
| **ACEi/ARB** | 1527.3 (27.5) | 626.7 (27.2) | 0.006 |
| **Diuretics** | 1382.2 (24.9) | 570.8 (24.7) | 0.002 |
| **Low income** | 789.7 (14.2) | 322.7 (14.0) | 0.006 |

Abbreviations: ACEi, angiotensin-converting enzyme inhibitor; AF, atrial fibrillation; ARB angiotensin receptor blocker; ASD, absolute standardized difference; CCB, calcium channel blocker; CCI, Charlson comorbidity index; COPD, chronic obstructive pulmonary disease; DHP, dihydropyridine; DBP, diastolic blood pressure; DOAC, direct oral anticoagulant; GFR, glomerular filtration rate; ICH, intracranial hemorrhage; IQR, interquartile range; SBP, systolic blood pressure; SD, standard deviation.

Group 3, early rhythm control (ERC) alone; and Group 4, both ERC and healthy lifestyle

**Supplementary Table 5. Weighted event numbers, incidence rates, and weighted hazard ratios for group 2 versus group 4 after propensity score weighting**

|  | **Group** | **Number** | **Weighted**  **event number** | **Weighted IR**  **(Per 100 PY)** | **IPTW**  **HR (95% CI)** | **p-value** |
| --- | --- | --- | --- | --- | --- | --- |
| **Ischemic stroke** | Group 2 | 8353 | 652 | 2.05 | 1 (reference) | 0.227 |
|  | Group 4 | 2296 | 155 | 1.84 | 0.898 (0.753-1.070) |  |
| **Heart failure** | Group 2 | 8353 | 546 | 1.70 | 1 (reference) | 0.494 |
|  | Group 4 | 2296 | 135 | 1.58 | 0.936 (0.775-1.131) |  |
| **Death** | Group 2 | 8353 | 1674 | 5.05 | 1 (reference) | 0.348 |
|  | Group 4 | 2296 | 412 | 4.71 | 0.950 (0.853-1.058) |  |
| **Composite outcome** | Group 2 | 8353 | 2373 | 7.70 | 1 (reference) | 0.072 |
|  | Group 4 | 2296 | 576 | 7.03 | 0.920 (0.840-1.008) |  |

Abbreviation: CI, confidence interval; HR, hazard ratio; IPTW, inverse probability of treatment weighting; IR, incidence rate; PY, person-year.

Group 2, healthy lifestyle (HLS) alone; and Group 4, both early rhythm control and HLS

**Supplementary Table 6. Weighted event numbers, incidence rates, and weighted hazard ratios for group 3 versus group 4 after propensity score weighting**

|  | **Group** | **Number** | **Weighted**  **event number** | **Weighted IR**  **(Per 100 PY)** | **IPTW**  **HR (95% CI)** | **p-value** |
| --- | --- | --- | --- | --- | --- | --- |
| **Ischemic stroke** | Group 3 | 5563 | 353 | 1.82 | 1 (reference) | 0.405 |
|  | Group 4 | 2308 | 162 | 1.97 | 1.082 (0.898-1.304) |  |
| **Heart failure** | Group 3 | 5563 | 427 | 2.20 | 1 (reference) | 0.440 |
|  | Group 4 | 2308 | 170 | 2.05 | 0.932 (0.780-1.114) |  |
| **Death** | Group 3 | 5563 | 1278 | 6.35 | 1 (reference) | <0.001 |
|  | Group 4 | 2308 | 437 | 5.10 | 0.801 (0.719-0.893) |  |
| **Composite outcome** | Group 3 | 5563 | 1706 | 9.09 | 1 (reference) | <0.001 |
|  | Group 4 | 2308 | 618 | 7.79 | 0.857 (0.782-0.940) |  |

Abbreviation: CI, confidence interval; HR, hazard ratio; IPTW, inverse probability of treatment weighting; IR, incidence rate; PY, person-year.

Group 3, early rhythm control (ERC) alone; and Group 4, both ERC and healthy lifestyle

**Supplementary Table 7. Subgroup analyses according to oral anticoagulant use**

| **Outcome** | **Subgroup** | **Group*** | **Number** | **Event** | **IR**  **(Per 100 PY)** | **Model 3**  **HR (95% CI)** | **p-value** | **p-for-interaction** |
| --- | --- | --- | --- | --- | --- | --- | --- | --- |
| **Ischemic stroke** | **No OAC** | **1** | 12740 | 590 | 1.24 | 1 (reference) | 0.042 | 0.014 |
|  |  | **2** | 3983 | 140 | 0.88 | 0.777 (0.645-0.936) |  |  |
|  |  | **3** | 2226 | 90 | 1.12 | 0.920 (0.736-1.151) |  |  |
|  |  | **4** | 798 | 25 | 0.82 | 0.763 (0.509-1.142) |  |  |
|  | **OAC** | **1** | 12353 | 1649 | 3.77 | 1 (reference) | <0.001 |  |
|  |  | **2** | 4368 | 503 | 3.13 | 0.897 (0.811-0.992) |  |  |
|  |  | **3** | 3339 | 266 | 2.34 | 0.655 (0.575-0.747) |  |  |
|  |  | **4** | 1508 | 121 | 2.29 | 0.680 (0.565-0.819) |  |  |
| **Heart failure** | **No OAC** | **1** | 12740 | 639 | 1.34 | 1 (reference) | 0.236 | 0.728 |
|  |  | **2** | 3983 | 144 | 0.91 | 0.847 (0.705-1.016) |  |  |
|  |  | **3** | 2226 | 110 | 1.38 | 1.031 (0.840-1.265) |  |  |
|  |  | **4** | 798 | 26 | 0.84 | 0.824 (0.554-1.225) |  |  |
|  | **OAC** | **1** | 12353 | 1399 | 3.17 | 1 (reference) | 0.108 |  |
|  |  | **2** | 4368 | 398 | 2.44 | 0.889 (0.794-0.994) |  |  |
|  |  | **3** | 3339 | 337 | 2.98 | 0.956 (0.848-1.078) |  |  |
|  |  | **4** | 1508 | 118 | 2.22 | 0.856 (0.708-1.035) |  |  |
| **Death** | **No OAC** | **1** | 12740 | 4308 | 8.84 | 1 (reference) | <0.001 | 0.598 |
|  |  | **2** | 3983 | 889 | 5.48 | 0.724 (0.673-0.778) |  |  |
|  |  | **3** | 2226 | 628 | 7.69 | 0.911 (0.837-0.992) |  |  |
|  |  | **4** | 798 | 150 | 4.80 | 0.631 (0.536-0.744) |  |  |
|  | **OAC** | **1** | 12353 | 3244 | 6.91 | 1 (reference) | <0.001 |  |
|  |  | **2** | 4368 | 807 | 4.72 | 0.747 (0.691-0.807) |  |  |
|  |  | **3** | 3339 | 688 | 5.78 | 0.914 (0.841-0.993) |  |  |
|  |  | **4** | 1508 | 236 | 4.25 | 0.718 (0.628-0.820) |  |  |
| **Composite outcome** | **No OAC** | **1** | 12740 | 4834 | 10.39 | 1 (reference) | <0.001 | 0.016 |
|  |  | **2** | 3983 | 1027 | 6.57 | 0.741 (0.692-0.794) |  |  |
|  |  | **3** | 2226 | 719 | 9.18 | 0.919 (0.849-0.994) |  |  |
|  |  | **4** | 798 | 178 | 5.87 | 0.661 (0.568-0.768) |  |  |
|  | **OAC** | **1** | 12353 | 4843 | 11.76 | 1 (reference) | <0.001 |  |
|  |  | **2** | 4368 | 1355 | 8.84 | 0.829 (0.780-0.880) |  |  |
|  |  | **3** | 3339 | 1033 | 9.53 | 0.859 (0.803-0.920) |  |  |
|  |  | **4** | 1508 | 376 | 7.41 | 0.729 (0.656-0.811) |  |  |

Abbreviation: CI, confidence interval; HR, hazard ratio; IR, incidence rate; OAC, oral anticoagulant; PY, person-year.

*Group 1, without both early rhythm control (ERC) and healthy lifestyle (HLS); Group 2, HLS alone; Group 3, ERC alone; and Group 4, both ERC and HLS
