# Supplementary figures and images for: Combination of early rhythm control and healthy lifestyle on the risk of stroke in elderly patients with new-onset atrial fibrillation: a nationwide population-based cohort study
Source: Front Cardiovasc Med. 2024 Feb 15;11:1346414. doi: 10.3389/fcvm.2024.1346414 (PMC10902049; doi:10.3389/fcvm.2024.1346414)

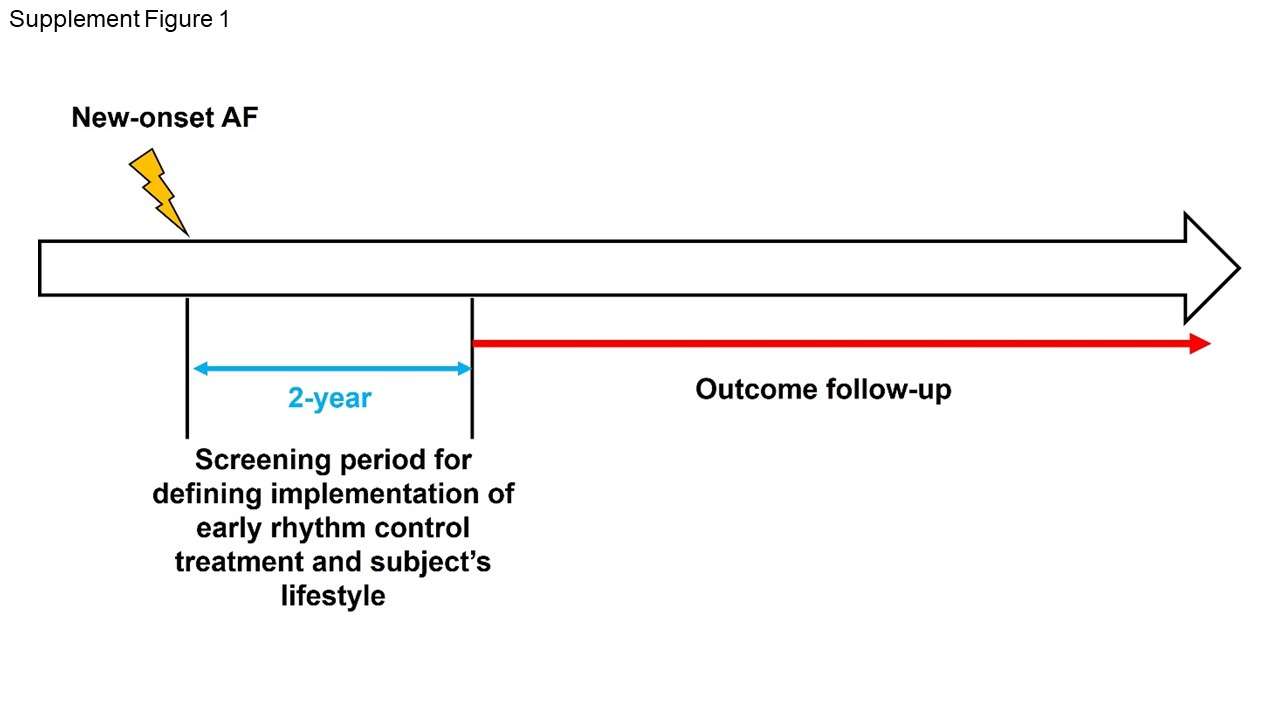

Supplement: Supplementary file 2 [file Image1.jpeg]
